# Molecular Mechanisms of Glaucoma Pathogenesis with Implications to Caveolin Adaptor Protein and Caveolin-Shp2 Axis

**DOI:** 10.14336/AD.2023.1012

**Published:** 2024-10-01

**Authors:** Mojdeh Abbasi, Vivek Gupta, Nitin Chitranshi, Petros Moustardas, Reza Ranjbaran, Stuart L. Graham

**Affiliations:** ^1^Macquarie Medical School, Faculty of Medicine, Health and Human Sciences, Macquarie University, North Ryde, Sydney, NSW 2109, Australia.; ^2^Division of Ophthalmology, Department of Biomedical and Clinical Sciences, Linköping University, Linköping Sweden.; ^3^Diagnostic Laboratory Sciences and Technology Research Center, School of Paramedical Sciences, Shiraz University of Medical Sciences, Shiraz, Iran

**Keywords:** retina, glaucoma, Caveolin, Shp2 phosphatase, IOP, RGCs

## Abstract

Glaucoma is a common retinal disorder characterized by progressive optic nerve damage, resulting in visual impairment and potential blindness. Elevated intraocular pressure (IOP) is a major risk factor, but some patients still experience disease progression despite IOP-lowering treatments. Genome-wide association studies have linked variations in the Caveolin1/2 (CAV-1/2) gene loci to glaucoma risk. Cav-1, a key protein in caveolae membrane invaginations, is involved in signaling pathways and its absence impairs retinal function. Recent research suggests that Cav-1 is implicated in modulating the BDNF/TrkB signaling pathway in retinal ganglion cells, which plays a critical role in retinal ganglion cell (RGC) health and protection against apoptosis. Understanding the interplay between these proteins could shed light on glaucoma pathogenesis and provide potential therapeutic targets.

## 1. Introduction

Glaucoma is a very common disorder in aging populations and is a leading cause of irreversible loss of vision around the world. The main pathological hallmark of this disease is a progressive loss of retinal ganglion cells (RGCs) and optic nerve structural damage with the major risk factor being enhanced intraocular pressure (IOP) [1]. Unfortunately, some patients demonstrate disease progression despite IOP-lowering management which is currently the primary treatment for glaucoma [2]. Therefore, unraveling the IOP-independent mechanisms remains a priority. Genome-wide association studies (GWAS) have linked variations in *Caveolin1/2 (CAV-1/2)* gene loci as a risk factor in glaucoma [3]. However, the biochemical role of Cav-1 and the potential mechanisms underlying this genetic association remains to be explored. Cav-1 is the prominent signature protein of caveolae membrane invaginations and is involved in a variety of signaling pathways [4]. The absence of Cav-1 has previously been demonstrated to impair retinal function, and our investigations established that it is implicated in the regulation of brain-derived neurotrophic factor (BDNF)/TrkB signaling in the RGCs through its interactions with a cytosolic phosphatase Shp2 [5, 6]. BDNF and its receptor, TrkB has been shown to play critical roles in maintaining the health of RGCs and protecting them from apoptosis [7]. In this review, we discuss the known mechanisms of retinal cell injury in glaucoma with a particular focus on an important molecular mechanism underlying BDNF/TrkB signaling mediated by Cav-1 and Shp2 phosphatase and its implications in glaucoma pathogenesis.

## 2. Retina

**Figure 1.**
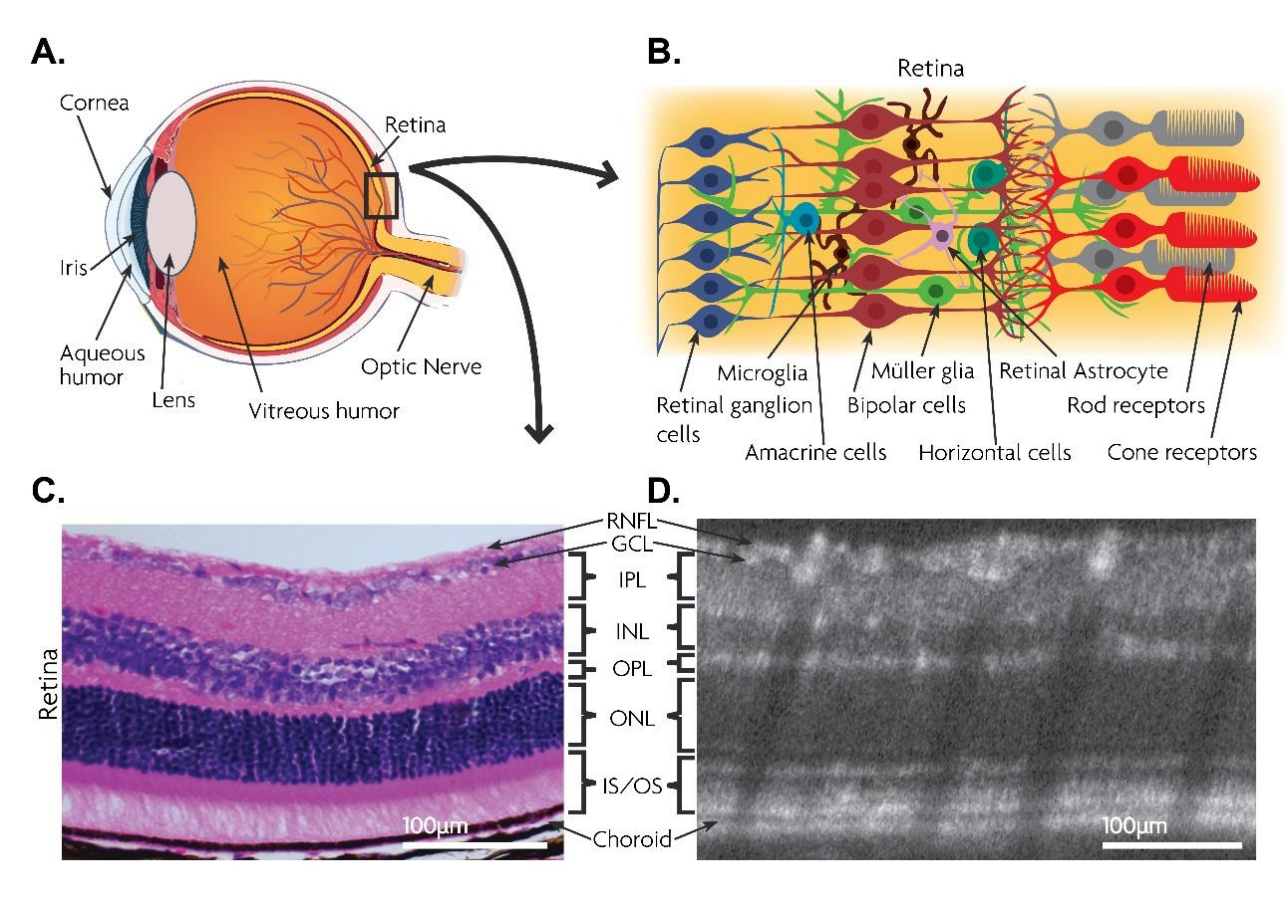
Anatomy of the eye and arrangement of different types of cells and layers in the retinal structure. Anatomical representation of the eye (A) and Schematic enlargement of retinal structure and the main cell types (B). Light enters the eye and the retina receives the image when it passes through the cornea. Photons are captured by photoreceptors and the signal then is transmitted to bipolar and amacrine cells after being converted to electric signals. Comparison of the histological image (Scale bar= 100 μm) (C) and *in vivo* spectral domain optical coherence tomography (SD-OCT) scan (Scale bar= 100 μm) (D) of the C57BL/6 wildtype mouse for retinal layers. Cross-section of the retina indicates the laminated structure which involves RNFL, retinal nerve fiber layer; GCL, ganglion cell layer axons of which converge in the optic nerve; IPL, inner plexiform layer; INL, inner nuclear layer containing bipolar, horizontal, and amacrine cells; OPL, outer plexiform layer; ONL, outer nuclear layer which contains the photoreceptor cells; IS/OS, inner and outer segments.

The retina is a neuro-ectodermal derivative of the brain which features several physiological, cellular, and biochemical similarities with brain tissue [8]. It consists of a multi-layered, highly organized tissue lining the posterior wall of the eye. The three distinct layers of the retina include the outer nuclear layer (ONL), the inner nuclear layer (INL), and the ganglion cell layer (GCL) separated by two plexiform layers [9] (Fig. 1). The innermost layer contains RGC neurons whose extra-ocular axons converge in the optic nerve extending towards the lateral geniculate nucleus (LGN), which is the primary visual relay center in the thalamus. Retina shares similarities with the brain and therefore, a number of diseases that impact the brain also have manifestations and symptoms in the ocular system [10]. Moreover, with the advancement of technology, subtle changes in both human and animal retinas can be directly imaged and assessed *in vivo*, and as such the retina is being extensively used as a means to study neurodegenerative disorders that can be vision-specific, such as glaucoma, age-related macular degeneration (AMD) but even not specific to the eyes, like Alzheimer’s disease (AD) which together impact a high percentage of the aging population [11].

## 3. Glaucoma

Historically categorized as a condition linked to elevated IOP, the term "glaucoma" has evolved to encompass a genetically heterogeneous group of chronic optic neuropathies, all sharing the common feature of slow and progressive neurodegeneration affecting RGCs and their associated axons [12]. The disease is the leading cause of irreversible blindness and is projected to affect more than 100 million individuals between 40 and 80 years of age by 2040 [13]. The disease is characterized by injury to the axons of RGCs and progressive degeneration of the somata and axons of these cells within the retina as well as Wallerian degeneration of the myelinated axons in the optic nerve (ON) [14]. Elevated IOP stands as the primary risk factor closely linked to glaucoma with the extent of this elevation being directly correlated with the severity of the RGC degeneration [15]. Essential to maintaining the intricate equilibrium of intraocular pressure within the eye is the aqueous humor production and drainage system. This equilibrium is upheld through the harmonious functioning of the conventional outflow (CO) pathways, encompassing the trabecular meshwork (TM) and Schlemm's canal (SC). These structures orchestrate the pace of aqueous humor drainage, thereby intricately governing intraocular pressure and finely tuning resistance to outflow. Disturbance of this delicate equilibrium can result in the malfunctioning of the drainage system, leading to an increase in IOP that exerts damage to the lamina cribrosa. This, in turn, affects the axons of RGCs as they pass through the optic nerve head, ultimately resulting in the degeneration of RGCs due to pressure-induced axonal damage [4, 15, 16]. Based on changes in the iridocorneal angle, glaucoma can be classified into two broad categories, primary open-angle glaucoma (POAG) and acute angle closure glaucoma. POAG is a complex disorder with a slowly progressive loss of visual sensitivity accounting for around 74% of glaucoma conditions [17]. POAG is going to be the main focus of the mechanisms discussed in this review. Acute angle-closure, on the other hand, has a sudden onset of symptoms due to immediate elevation of IOP. This type is responsible for less than 10% of glaucoma cases [18], though, it is also a major cause of morbidity [19]. Epidemiologic studies demonstrated that the prevalence of POAG, although varying between populations, was nearly 64.3 million in 2013 which is estimated to increase to 111.8 million patients worldwide by 2040 [13]. Several clinical studies have shown that the most prominent risk factor associated with the onset and progression of glaucoma is elevated intraocular pressure. Not all glaucoma patients demonstrate increasing IOP, though, with 25% to 50% of patients presenting with an IOP within the normal range, a condition termed normal tension glaucoma (NTG) [18]. Progression can still occur even subsequent to adequate lowering of IOP via medication or surgery treatments [20]. Additional risk factors may include age, race, family history, vascular dysregulation, myopia, and relatively thin corneal thickness. Other risk factors of lesser positive correlation with the disease include diabetes mellitus and systemic hypertension [21]. Current evidence in the literature does support the notion that either systemic hypertension or hypotension could be considered as risk factor for glaucoma [22] although the precise association remains controversial. Apart from non-genetic components, the familial nature of POAG has been shown to play a role in POAG pathogenesis suggesting that genetic factors contribute to the pathogenesis of glaucoma [23]. The inheritance of glaucoma is complex in most cases, where the interaction of either gene variants or environmental factors seems to influence the emergence of the disease [17, 24]. However, linkage analysis, a tool for estimating the genetic mapping of a particular trait or phenotype within a family group [25] demonstrated that the mendelian inheritance in POAG accounts for only around 5% of all glaucoma cases. This technique has led to the discovery of at least 20 genetic loci associated with POAG in family-based studies including myocilin (MYOC), Optineurin (OPTN), TANK binding kinase 1 (TBK1), and NTF4 to name a few [17]. Mutation in *MYOC* and *OPTN*, the high penetrance glaucoma genes, is responsible for about 4% of POAG conditions globally whereas the remaining is likely attributed to a combination of both environmental and genetic factors contributing to the complex inheritance of glaucoma [26]. Some environmental factors include nutritional factors or some types of physical exercise although further research is required to precisely outline their roles in IOP elevation [12, 27]. Also, a gene-environment interaction was detected between nitric oxide synthetase 3 (NOS3) SNPs and postmenopausal hormone therapy in women in relation to high-tension POAG [27]. GWAS have also identified additional genetic loci which are associated with either glaucoma or a high level of IOP. In particular, a number of studies that studied populations of different countries have resulted in the discovery of several associations between specific genes and POAG including CDKN2B-AS1, TMCO1, CAV1/CAV2, SIX1/SIX6, ABCA1, TGFBR3, and many other additional loci, [28] although detailed discussions of each are beyond the scope of this review. In 2010 Thorleifsson et al., for the first time mapped a susceptibility variant (rs4236601) as a new genetic risk factor on chromosome 7q31 which was associated with POAG [3]. This area contains two genes, *CAV-1* and *CAV-2*, genes that encode for the proteins Caveolin 1 and 2, respectively. Later, several population-based studies provided evidence of *CAV-1* association in glaucoma pathogenesis although the significance varies among the population groups [4, 29-31]. Caveolin is the principal component of membrane lipid rafts caveolae, ubiquitously expressed in all mammalian cells including the majority of retinal cells and TM [4, 32]. This protein has proven functions in a variety of signaling pathways in transcellular transport and mechano-transduction [33]. Additionally, GWAS has been applied to explore the association of this gene with glaucoma-associated quantitative traits such as IOP, optic cup-disc ratio, and central cornea thickness [17]. *CAV-1/2* was also later addressed to be one of the IOP-associated genetic loci [34, 35]. Even though several GWAS have reproducibly implicated the association of Cav-1 polymorphisms with POAG and other ocular hypertension metrics [3, 36-38], the limitations of GWAS should be considered in all future studies to enhance the statistical power enabling the actual and not misleading associations [39, 40].

GWAS is a powerful and efficient method for detecting potential disease-related risk variants. However, there are certain potential confounding factors that may mask the extent of statistical association. Confounding might be due to genetic effects which is population based or heterogeneity because of various patterns of linkage disequilibrium (LD). The inconsistent results of GWAS may also stem from the interference of environmental factors, disease status, population-related disease rate, and ancestral origin of the locus [39, 41]. Also, some large-scale GWAS are only restricted to specific ethnicity or population groups which may restrict the generalizability of the results. Accordingly, efforts towards more inclusive GWAS investigations using more diverse populations will aid in progressively overcoming this limitation [40].

## 4. Neuroprotection strategies

The primary goal of all glaucoma treatment strategies is to prevent further optic neuropathy and to slow down the loss of the shrinkage of the visual field [18]. As POAG is mainly diagnosed with significantly increased IOP levels in patients, the exclusive evidence-based therapy currently available for the patients is either pharmacological or surgical approaches for IOP lowering, and towards a target level of IOP at which the rate of glaucoma progression is sufficiently decelerated to avoid extra visual impairment [42]. Although various clinical trials illustrated that IOP lowering and adequate pressure control might be able to substantially reduce the progression of the disease in many patients even in advanced stages, it is still inadequate for some patients who continue to develop progressive visual impairment [43]. A significant reason for the absence of proper alternative treatment strategies is that there is an incomplete understanding of either the molecular basis of the disease or how it affects the retinal structure and function [44]. This paradigm highlights the role of IOP-independent mechanisms in mediating RGC degeneration which should be considered in future treatment strategies. In recent years, several new treatment strategies have been proposed and investigated in preclinical models focusing on RGC neuroprotection through applying the pharmacological approach to protect against optic nerve damage and RGC apoptosis. This includes a variety of neuroprotectors such as anti-apoptotic agents [45], neurotrophic factors [46], Nitric oxide synthase antagonists [47], and antioxidants [48]. Most recently, gene therapy approaches [49], and stem cell transplantation therapy [50], have been attempted. It is hoped that the latter might be used as a source of cell replacement for lost RGCs or even to carry and express the essential neuroprotective genes to protect them against glaucoma injury. Targeting the intracellular intermediates to prevent RGC loss can be regarded as an alternative strategy to help patients [51]. Several important potential interpretations proposed to trigger RGC death in glaucoma include glial cell activation, ischemia, oxidative stress, endoplasmic reticulum (ER) stress, and neurotrophic deprivation are discussed below.

### 4.1. Glial cell activation and RGC death

The retina, a highly organized laminar structure, consists of neurons and glial cells. There are three types of glial cells in the retina including microglia, astrocytes, and Müller cells. The role of glial cells in supporting the RGCs is complex. Under normal physiological conditions, these cells protect the RGCs through providing metabolic and neuroprotective support as well as sustaining the homeostasis of the extracellular matrix of these cells [46, 52]. However, upon exposure to high IOP injury, glial cells are activated and trigger RGCs apoptosis through a variety of mechanisms such as secreting elevated levels of tumor necrosis factor alpha (TNF-a) or nitric oxide (NO) which activate the apoptotic pathway in RGCs with resultant cell death [53].

### 4.2. Ischemia and oxidative stress

Significant elevation of IOP might also contribute to ischemia leading to the absence of sufficient oxygen and nutrient withdrawal in the retina. In normal conditions, the perfusion pressure (arterial blood pressure minus IOP) and therefore, the blood flow is preserved within the normal range. In high IOP, vascular supply is impaired, and deficiency of optic nerve blood flow causes ischemia [54]. Moreover, some studies have reported the disease pathogenesis with a high level of reactive oxygen species (ROS) production in the retina when it is exposed to glaucomatous conditions. This condition of oxidative stress is an indicator of insufficient antioxidant enzymes to protect against this type of injury. Therefore, in glaucoma, there is an excessive generation of ROS, and this disturbs the metabolic process through damaging the DNA, lipids, and protein in cells, which ultimately affects RGC viability and function [55].

### 4.3. Endoplasmic reticulum stress and RGC injury

The endoplasmic reticulum (ER) is an intracellular organelle with essential roles in biosynthesis, posttranslational modification, folding, and trafficking of newly synthesized proteins. In addition, only the properly-folded proteins are released from the ER and transferred to the Golgi apparatus for further processing [56]. Any type of injury or stress perturbing the ER function results in ER-stress which stimulates the activation of unfolded protein response (UPR); a pathway that regulates ER homeostasis and protects the cell against misfolded protein aggregation and cytotoxic damage through ubiquitination and clearance of misfolded protein [57]. However, when the ER function is severely disturbed and in cases of sustained or overwhelmed UPR, a large number of apoptotic controlling intermediates are activated that stimulate the apoptotic cascade and ultimately result in the apoptotic death of the cell [56]. One study has addressed the ER-mediated apoptosis of the RGCs subsequent to neurotrophin withdrawal, ischemia, and ocular hypertension in animal models of glaucoma [51, 58]. Raised IOP results in apoptosis activation in RGC cells which is accompanied by increased levels of ER-related stress markers including binding immunoglobulin protein (BiP), protein kinase RNA (PKR)-like ER kinase (PERK) and C/EBP homologous protein (CHOP) [59]. Therefore, further understanding of the role played by ER in glaucoma-induced RGC death could provide insights to consider the ER stress pathway as a potential target for neuroprotection in glaucoma.

### 4.4. Neurotrophin deprivation

Neurotrophins (NTs) are secreted proteins that regulate neural growth, differentiation, and function through hindering the apoptotic activation in the cells. The NT family comprises nerve growth factor (NGF), brain-derived neurotrophic factor (BDNF), NT-3, and NT-4/5. NTs play their roles through two distinct receptors of either receptor tyrosine receptor kinase (Trk) or the P75NT receptors (P75NTR) which upon binding mediate the survival or apoptosis downstream signaling respectively [60]. NGF has a high affinity for TrkA while BDNF and NT4/5 preferentially bind to TrkB and NT3 to TrkC. The Trk receptor family holds various physiological roles in various tissues and has an indispensable role in signal transmission for neural development and survival [61]. On the contrary, P75NTR is a quite complex receptor belonging to the tumor necrosis factor (TNF) family, and once activated it is coupled to the mitochondrial apoptotic cascade [62]. The prominent source of NTs for the retina is locally produced in the brain and retrogradely transported to carry out the survival signaling in the retina [63]. Upon NT binding to the Trk receptor, they are activated, internalized and retrogradely transferred to the axons and ultimately reach the ganglion cell bodies where they mediate the survival signaling [45]. Paradoxically if NT precursors are secreted in a complex with P75NTR, the receptor can serve as a death signal mediating apoptosis [62]. The decline or absence of neurotrophins such as BDNF has been widely proposed to stimulate the onset of RGC apoptosis which ultimately results in the progressive degeneration of cells. It is believed that upon being exposed to elevated IOP the retrograde supply of NTs through RGC axons is obstructed resulting in a notable decline in survival signaling and thereby activating the intrinsic apoptotic pathway [64, 65]. However, whether this process is directly caused by the loss of NTs or through the effects of some other molecular degenerative changes remains undetermined.

Therefore, providing an exogenous source of NTs (BDNF in particular) or NT gene therapy has the potential to be an effective strategy for protecting ganglion cells against high IOP-mediated damage. However, a number of studies demonstrated that NT delivery such as BDNF either through repeated intravitreal injection [49, 66, 67] or genetic therapy treatments [68] could only transiently delay the death of RGCs providing short-term RGC protection and ultimately the cells succumb to the degenerative cascade [69, 70]. This might be at least in part due to the absence of a sufficient level of Trk receptor in response to exogenous NT supply [71]. Additionally, it is believed that RGCs might become insensitive to the NT-stimulating effect while also the exclusively enhanced level of NTs might not be enough to stimulate ganglion cell survival [72, 73]. Sustained survival of RGCs remains the current goal of all neuroprotective strategies although the response of these cells to NTs under high IOP conditions is quite complex. Consequently, further understanding of the mechanisms underlying cell survival, as well as death pathways, will assist us in providing long-term RGC protection.

Regardless of the mechanism underlying RGC damage, it is increasingly established that most cells are eliminated through apoptosis; the default programmed intrinsic cell death [51, 52]. A number of studies have evidenced that a similar feature of death has been observed in RGCs following experimentally induced models of glaucoma, optic nerve crush, and human glaucoma highlighting that loss of RGCs in this disease occurs via apoptosis [74, 75]. In glaucoma, based on the type of death signal, apoptosis is shown to be activated either extrinsically or intrinsically through TNF and neurotrophin deprivation respectively [76]. Suppressing the apoptotic process of RGC degeneration, however, resulted in a significant delay in cell death and imparted protection to the dying RGCs following various models of experimental glaucoma [69].

### 4.4.1. BDNF/TrkB signaling

Amongst all the neurotrophins, BDNF in particular is a potent neurotrophin that plays a key role in maintaining the health of RGCs and preserving them from apoptosis [59, 77]. BDNF is well expressed in the superior colliculus, RGCs, and inner nuclear cells and has been demonstrated to primarily exert its biological effects through binding with its high-affinity receptor, TrkB [78]. TrkB is also highly expressed in the RGC dendrites and cell bodies [79]. Although the primary source of BDNF supply for RGCs is known to be the brain, studies have also demonstrated that there is a level of local expression of this neurotrophin in the RGCs, Müller glial cells, and astrocytes [80]. Following BDNF binding, TrkB is activated through dimerization and autophosphorylation on its critical intracellular tyrosine residues [61]. BDNF binding to its cognate receptor will result in the initiation of cell survival signaling pathways important of which are mitogen-activated protein kinase/extracellular signal-regulated kinase 1 and 2 (MAPK/Erk1/2) and phosphatidylinositol-3 kinase (PI3K/AKT) downstream signaling pathways [81]. ERK and AKT have critical roles in promoting cell proliferation, differentiation, and survival. However, MAPK/Erk has been proposed to make more substantial contributions to adult RGC survival in response to BDNF-TrkB stimulation compared to AKT signaling pathways. This is due to the fact that selective inhibition of ERK cascade significantly reduced the TrkB survival signaling while no effects were observed following PI3K inhibition. This strongly suggests that ERK but not AKT pathway may be critical for RGC survival [82, 83]. As mentioned previously, BDNF and TrkB gene therapy is a promising strategy which is believed to provide neuroprotection for RGCs subsequent to high IOP injury although it only has transient effects. Therefore, the exact mechanisms underlying the sensitivity of either BDNF or TrkB molecules and the related signaling intermediates under elevated IOP conditions require further investigation. Recent studies have established that BDNF/trkB signaling in the RGCs is negatively regulated by one of the TrkB interactive proteins, SH2 domain tyrosine phosphatase PTPN11 (Shp2) [60, 84]. Interestingly, the activation of Shp2 is shown to be mediated through its interactions with the adapter protein Cav-1, the gene described as a risk factor for glaucoma [3]. We also more precisely evidenced that the negative association of Shp2 phosphatase and BDNF/TrkB signaling provides an *in vivo* protective effect under chronic experimental glaucoma conditions and it is effectively dependent on the Cav-1 protein [5]. The precise molecular mechanism and role of the Shp2 phosphatase and the Cav-1 adaptor protein will be discussed in detail in the following sections.

### 4.4.2. The SH2 domain-containing tyrosine phosphatase

Activation of downstream survival pathways including ERK and AKT requires the interaction of various proteins with TrkB [85], creating a tandem phosphorylation and dephosphorylation signaling route. One of the TrkB interactive proteins is the Shp2 phosphatase, which has been identified as an essential intermediate in the BDNF-mediated MAPK [84]. Shp2 is a 593 amino acid non-transmembrane protein tyrosine phosphatase (PTP) encoded by the *PTPN11* gene [86]. This phosphatase is ubiquitously expressed and involves the Src homology 2 (SH2) domain facilitating its interactions with phospholipids and phosphoproteins in response to endogenous ligands such as hormones, growth factors, and cytokines [87]. Shp2 plays prominent biological roles in regulating several signal transduction cascades affiliated with its function in the early development of vertebrates, cell proliferation, differentiation, transcription regulation, and metabolic control [60]. Shp2 dysregulation has been stated to be associated with cardiovascular [88] as well as neurodegenerative disorders of the brain and the eye [89, 90]. In the retina, Shp2 is well expressed in the GCL and INL, and its reactivity has been detected in photoreceptors [84]. Shp2 is suggested to be involved in neuronal morphogenesis during early embryonic stages while during postnatal development no further deficits in retinal differentiation were observed in Shp2 mutants indicating a critical role of the protein during early retinal development [91]. In Shp2 ablated rodent models, retinal degenerative changes are documented, that mainly localize to the inner retina along with optic nerve atrophy, reinforcing the vital role played by Shp2 in the retina [92, 93]. As previously discussed, the neuromodulatory effects of BDNF play an essential role in maintaining the health of RGCs and protecting them from apoptosis [72, 94]. BDNF induces Shp2 phosphorylation and its subsequent association with adaptor proteins GRB2/SOS for complete MAPK activation [61, 95] which is shown to be neuroprotective in glaucoma conditions [93]. A consensus amino acid sequence (NPXY motif) in the TrkB sequence is recognized by the phosphotyrosine binding (PTB) domain of Shp2 [95]. This interaction helps in mediating the pathway in a BDNF-dependent manner. Shp2 may thus function as a transducing protein connecting BDNF and TrkB to MAPK activation. This suggests a positive role of Shp2 in regulating the MAPK signaling pathway [60, 96]. Depending on the partners and downstream signaling pathways, Shp2 phosphatase has also been shown to exert dominant regulatory negative effects on BDNF signaling [97]. We previously identified the negative effects of Shp2 on BDNF/TrkB activation in the RGCs isolated from rodent eyes following optic nerve axotomy. An increased Shp2-TrkB interaction was observed under glaucomatous stress conditions indicating a pathological crosstalk between the two proteins while its inhibition restored TrkB activity under the same condition [84, 98]. TrkB activation has been shown to play a critical role in RGC survival under various stress conditions and therefore Shp2 activation or its enhanced interactions with TrkB are likely to suppress the neuroprotective pathways, and lead to RGC loss and optic nerve axonal deterioration [84]. Our studies have also indicated that Shp2 overexpression in both the neuroblastoma cell line SHSY5Y *in vitro* and in the RGCs *in vivo* leads to enhanced endoplasmic stress response induction and diminished TrkB activity [99]. Overall, these studies might explain the transient effects of TrkB or BDNF modulation in delaying the death of RGC under glaucomatous or stress conditions. BDNF/TrkB is a potent survival pathway in the visual system. Therefore, the negative regulation of TrkB through Shp2 phosphatase might explain why BDNF/TrkB activation in RGCs has only transient protective effects *in vivo*.

## 5. Caveolin and Caveola

### 5.1. General overview

**Figure 2.**
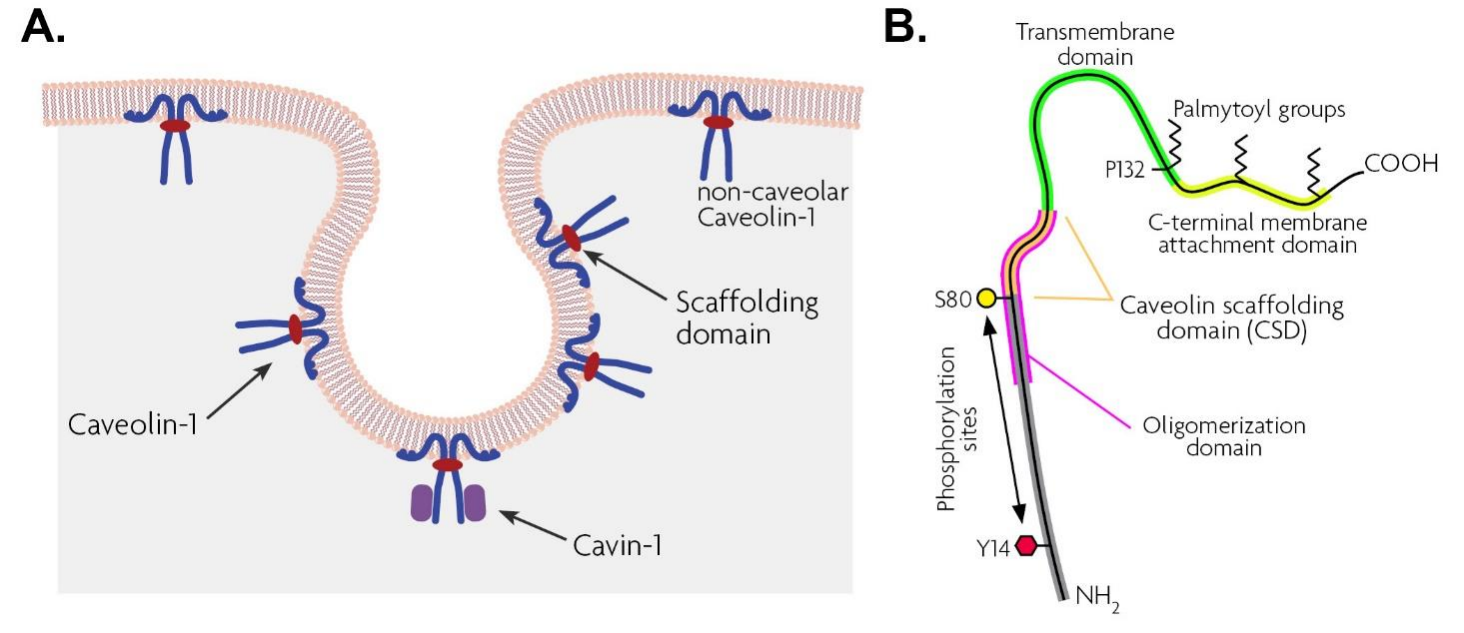
Structure of the caveola invaginations and caveolin ribbon diagram. (A) Schematic illustrations of caveola invaginations in the cell membrane indicating Cav-1 in the caveola organelle. Cavin-1 protein is shown in red which is required for stabilizing mature Caveolins and caveolae formation. (B) Ribbon-diagram overviews of A Cav-1 indicating N- and C-terminal as well as transmembrane domain (green) and Tyrosine 14 the phosphorylation domain of Cav-1.

Caveolae are 50-100 nm flask-shaped specialized membrane lipid rafts observed in different cell types such as vascular endothelium, fibroblasts, and adipocytes (Fig. 2). These membrane invaginations demonstrated to play critical roles in endocytosis, transcellular transport, cellular homeostasis, mechano-transduction, vesicular trafficking [33, 100], and apoptosis [101] while they are also implicated to be important in regulating several signal transduction pathways [102]. Caveolin is the principle structural constituent of caveolae, with three isoforms in vertebrates including Cav-1, 2, and 3. The scaffolding protein encoded by the *CAV-1* gene is a 22-KDa protein that behaves as an intrinsic adaptor protein with both N- and C-terminal facing the cytosol. This adaptor protein holds a highly conserved caveolin scaffolding domain (CSD) and also a phosphorylation site (Tyr14) which are implicated in mediating various signaling through facilitating the interaction between Caveolin and other intermediates [103, 104] (Fig. 2). Cav-1 and Cav-2 are nearly ubiquitously expressed in all non-muscle cells and researchers trying to generate cav knockout (KO), noticed that Cav-1 but not Cav-2 is indispensable for caveolae formation [105, 106]. However, studies have shown that caveola are less abundant or even not detected in neurons and it is demonstrated that Cav-1 undertakes a distinct role in neurons, separate from its conventional caveolae-related function and therefore additional research is needed to elucidate the roles of Cav-1 in overseeing cellular mechanisms, encompassing both caveolar and non-caveolar domains [4, 107]. Global genetic ablation of Cav-1 leads to the absence of morphologically identifiable caveolae in their tissues and the Cav-1 null mice generated are viable but demonstrate evidence of abnormalities such as hyperproliferative and vascular abnormalities, higher vascular permeability, perturbed lipid metabolism, and insulin resistance [108]. Additionally, dysfunction of Cav-1 has been associated with various diseases, such as Alzheimer’s disease, Parkinson’s disease as well as accelerated aging [109, 110]. It is clear that studying these animal models will assist in further understanding the mechanisms underlying these disorders and the involvement of caveolins and caveolae structures in these pathologies.

To function as a mediator in signal transductions, Cav-1 has interactions with a variety of signaling intermediates especially via tyrosine kinase receptors [4, 111]. This adaptor protein also interacts with the transporter, signaling molecules, G proteins, non-receptor protein kinases (Src family, protein kinase C), receptor tyrosine kinases (EGFR, VEGFR), Ras and enzymes such as endothelial nitric oxide synthase [112, 113]. These interactions can either positively or negatively mediate cellular signaling pathways [104]. The main functional Cav-1 domain, CSD, tends to behave as a suppressor of the Cav-1 interactive partners via direct physical interactions and therefore negatively regulate the activity of several growth pathways [6, 29]. For instance, there are several studies which addressed the CSD involvement in the negative regulation of endothelial nitric oxide synthase (eNOS) in vascular endothelium while the absence of Cav-1 results in enhanced nitric oxide (NO) production [114, 115]. The phosphorylation of the tyrosine residue 14 (Tyr14) of Cav-1, however, is suggested to confer positive stimulatory effects through binding to SH2 domain-containing proteins [33, 116]. Phosphorylation of Cav-1 in Tyr14 and transcriptional regulation of its expression may also have a potential role in glaucoma pathogenesis and TM cells [84, 117]. More specifically caveola and Caveolin functional domains are suggested to act as a signaling platform. Tyr14 phosphorylation of Cav-1 plays an important role in providing a docking site to anchor various signaling intermediates so that they would be able to perform their function in the membrane and consequently regulate several downstream signaling cascades [33, 118]. It is believed that the regulation of MAPK by Cav-1, through which Caveolin is thought to mediate the cell cycle and tumor progression, is of high importance. MAPKs are the downstream effectors of various signaling pathways and influence numerous cellular processes [119]. Upregulation of Cav-1, indeed, inhibits the MEK/ERK effectors, which is shown to be dependent on the CSD domain [108]. Accordingly, Cav-1 deficient mice exhibited a high level of ERK activation in fibroblasts, cardiac tissue as well as in hippocampus neurons [120]. Due to the regulatory effects of caveola and caveolins on intracellular signaling and their components, it is not very surprising that any change in caveola structure and caveolin expression level results in disease conditions as earlier discussed.

### 5.2 Expression of Cav-1 in the retina and functional consequences of Cav-1 deficiency

Several reports have addressed the immunoreactivity of Cav-1 in multiple ocular tissues including retinal layers and various retinal cell types such as retinal pigment epithelium, photoreceptors, Müller glial cells, and vascular endothelial cells [30, 121]. This protein is also expressed in ciliary muscle, trabecular meshwork, and Schlemm’s canal (SC) which might imply its function in the ocular system [4, 122]. Cav-1 appears to play an important role in the development stages of the retina while its depletion is shown to be associated with either pre or postnatal developmental deficits [6, 112, 123]. Still, its precise role in normal visual conditions remains largely unelucidated. Expression of Cav-1 in the ganglion cell layer, specifically, is in agreement with previously reported GWAS regarding the potential role of this protein in glaucoma pathogenesis [112, 124]. Based on this, manipulating the expression of this gene in different retinal cells will assist in further discovering the cell-specific function of the protein under normal and various stress circumstances. Localization of morphological caveolar structures and high immunoreactivity of caveolins in the retina, imply the critical association of caveolins to the retinal function. Li et al. (2012) demonstrated that Cav-1 ablated mice exhibit retinal functional deficits and vascular pathologies linked with reduced electroretinogram (ERG) measurement [6]. Consistent with this, in a more recent study we also identified that more specifically, these mice have reduced positive scotopic threshold (pSTR) amplitudes which are associated with their inner retinal function [30, 125]. Intriguingly, the functional implications were not supported by structural alterations in Cav-1 ablated animals [6, 125]. This led to a series of hypotheses that firstly, Cav-1 ablation might indirectly result in impaired Müller glial cell function. As Müller glial cells play prominent roles in neuroprotection, neural regeneration, and biochemical scaffolding of the retina [126, 127], their impaired function could underline our functional findings. Alternatively, this might be due to the compromised retinal microenvironment associated with perturbed blood-retinal barrier (BRB) integrity which resulted in retinal function deficits [128]. The BRB, located in the inner retina, consists of an interface between the neuroretina and the circulatory system and plays a critical role in maintaining retinal homeostasis [129]. Cav-1 has been implicated in supporting BRB maintenance in normal and disease conditions. In Cav-1 ablated mice, however, it is likely that the reduced number of caveolae contributed to relatively permeable BRB and ultimate pathological BRB breakdown [128] which resulted in the eventual loss of function in the retina. Consistent with this, a number of studies also found a notable increase in caveola and the Cav-1 protein, in hyper-permeable diabetic retinal vasculature [130]. In a ground-breaking recent study, Li and colleagues provided compelling evidence that the compound acetonide mitigates the loss of RGCs and reduces oxidative stress through the activation of the PI3K/AKT signaling pathway by enhancing the expression of Cav-1 which more specifically further highlights the potential of Cav-1 as a promising endogenous molecule whose impaired function plays a critical role in optic neuropathy and the degeneration of RGCs in glaucoma [131]. Another consequence of Cav-1 deficiency in Cav-1 ablated mice retinas is the increase in eNOS activity. Cav-1 has been shown to negatively regulate the activity of eNOS [4]. NO has critical physiological effects in mediating ocular hemodynamics, maintaining normal vasculature response and cell viability as well as protecting endothelial cells or fibers against pathophysiological conditions such as ischemia, glaucoma, and diabetes [132]. Under basal conditions binding of eNOS with the CSD domain of Cav-1 suppresses the enzymatic activity. Absence of Cav-1, nevertheless, leads to excessive eNOS activity resulting in cytotoxicity, loss of vascular tone, glial-cell mediated apoptosis, and RGC while blocking NO production has been directly correlated with a significant decline in RGC degeneration in experimental glaucoma model in rats [103, 133]. Alterations of caveolae and caveolin have also been linked to other ocular diseases such as diabetic retinopathy and inflamed uveitis retinas which make the KO animals a suitable model to further explore the function of this protein and their associations with these diseases [128].

### 5.3. Caveolins/caveolae in POAG and IOP

As discussed previously, the main feature of glaucoma is progressive damage to ganglion cell neurons and optic nerve fibers which is primarily attributed to elevated IOP [134]. The balance between aqueous humor production and drainage results in IOP maintenance [135] which is mainly performed through conventional structural pathways (trabecular meshwork and Schlemm’s canal) and uveoscleral outflow tracts [136]. Along with IOP and multifactorial risk factors, there are also critical genetic variants linked with this disease. Several GWAS studies strongly linked a risk variant (rs4236601) on a region encoded *Cav-1/2* genes as a risk factor in glaucoma pathogenesis [3, 128, 137]. More importantly, the same SNPs were also significantly associated with high-tension glaucoma and elevated IOP suggesting a role for Caveolins in aqueous humor outflow regulation [34, 138]. Caveolae and its main molecular component, Cav-1 abundantly exist in TM and SC endothelium and play prominent modulatory roles in maintaining IOP and aqueous humor outflow and it is likely that under normal conditions, the caveola protects the SC against the elevated level of IOP and nitric oxide signaling [106]. Interestingly, in another study silencing *CAV-1* resulted in higher aqueous humor drainage [139] while the global deletion of this gene led to increased outflow resistance and the animals had elevated ocular pressure [30, 106]. The differential consequences of Cav-1 silence on IOP in the first study were attributed to a temporary cellular response to the transient absence of Cav-1 protein. However, no significant changes were observed in the development or conventional outflow tissues in the Cav-1^-/-^ mice explored by various ultrastructure analyses [33, 106] which highlights the need for further investigations to unravel the underlying mechanism. Vascular tone abnormality is another risk factor for POAG with links to Cav-1. Interestingly, the *CAV-1* gene as a vascular tone regulator [140] showed a higher level of association with patients with glaucomatous paracentral visual field loss who were more likely to have systemic vascular dysfunction [38, 141]. This is consistent with a retinal vascular phenotype and systemic vascular alterations observed in Cav-1^-/-^ mice [142]. Table 1 lists the various studies on Caveolin with its association with glaucoma and IOP under normal and glaucoma conditions.

### 5.4. Other caveolae-associated proteins

Although in the absence of Cav-1, no morphologically identifiable caveola is produced, this protein is not sufficient for caveolae biogenesis, and other types of cytoplasmic proteins, Cavins, are essential for caveolae formation [143, 144] (Figure 2). Cavins have four subtypes with only Cavin-1 being crucial for caveolae biogenesis and its expression varies under different physiological conditions [145, 146]. Cavin-1, originally named “polymerase I and transcript release factor” (PTRF) due to its related function in the transcription process, is necessary for stabilizing mature caveolins while its absence triggers destabilization and proteolytically degradation of caveolins [147, 148]. Similar to Cav-1, Cavin-1 is also well expressed in the retina including GCL, trabecular meshwork, and SC [106]. In a study performed by Hauck (2010) who reported a quantitative proteome screening of differential expression of the retinal-associated protein in the uveitis disease, both Cav-1 and Cavin-1 dramatically upregulated in the retina, specifically in Müller glial cells [149]. This might partially explain the reason why cells like the Müller glial cell which do not possess caveolae under normal physiological conditions, still have the expression of extra-caveolar Cav-1 [150, 151]. Therefore, it is likely that under stress or disease conditions, Müller cells may need to build caveola, but this remains to be further explored [33]. Still, Cavins have not yet received vigorous attention in the visual system and there is a lack of knowledge about the role of this protein in retinal-associated diseases such as glaucoma.

**Table 1:** The table illustrates different studies Cav-1 studies and its association with glaucoma and IOP under normal and glaucoma conditions.

Animal/human model	Glaucoma model	Cav-1 function	Association with glaucoma	Refs.
POAG human eyes	POAG	Cav-1 phosphorylation (Tyr14) and transcriptional regulation play a role in glaucomatous alterations in TM cells.	Alteration in transcription regulation of Cav-1/pCav-1 (Tyr14) in glaucomatous TM tissue	[158]
Cav-1-/- mice	Normal condition	Cav-1 deficiency results in alteration in the subretinal microenvironment.	Reduced inner and outer retinal function	[6]
Rats AON-mediated Cav-1 silencing	Optic nerve axotomy/chronic	Cav-1 interacts with Shp2 and is phosphorylated in stressed RGCs.	Play a role in negative regulation of TrkB survival signaling by Shp2 phosphatase.	[84]
Human TM tissue (Lentivirus mediated Cav-1 silencing)	Normal condition	Increase in outflow rates	Cav-1 dysregulation resulted in ECM pathological changes observed in glaucoma.	[139]
Cav-1-/- mice	Normal condition	Cav-1 ablation reduces its interaction with eNOS and subsequent less inhibitory effects.	Reduced conventional outflow facility and enhanced eNOs activity	[115]
Cav-1-/- mice	Normal condition	Cav-1 deficiency results in a decrease in conventional drainage by around 48%.	Cav-1 is a key component for proper aqueous humor outflow.	[162]
Cav-1-/- mice	AOH (50 mmHg)	Cav-1 plays a role in IOP maintenance via modulation of aqueous humor drainage.	Caveolae protect the outflow pathway from AOH in vivo.	[106]
Cav-1-/- mice	Normal condition	Cav-1 is a negative regulator of eNOS while eNOS activation reduces IOP and increases conventional outflow. Caveolae play a critical role in the ability of outflow tissue to sense and respond to fluctuating IOP.	Loss of Cav-1 results in significant decline in pressure-dependent aqueous humor drainage and caveolae contribute to mechanoprotection. Thus, outflow cells lacking caveolae may exhibit compromised capacity to resist pressure-induced variations in mechanical load across the SC inner wall.	[106]
Cav-1-/- mice	AOH (110 mmHg)	Cav-1 deficiency exacerbated the lesion in the retina in AOH	Neuroprotective effect of Cav-1 on acute ocular hypertension	[121]
POAG human eyes	POAG	IOP regulation	Fewer Caveola on the plasma membrane of patients’ TM is associated with IOP dysregulation in glaucomatous TM tissue.	[163]
Mouse with Cav-1 depletion in endothelial cells (Cav-1ΔEC)	Normal condition	Endothelial Cav-1 deletion caused eNOS hyperactivity, enlargement of drainage vessels.	Tissue-specific expression of Cav-1 is essential for NO dispersion and the subsequent effect on IOP. Within SC and distal vessels Caveolae regulate IOP and pressure dependent CO through eNOS signaling.	[164]
Cav-1-/- mice	Chronic and acute rodent models of experimental glaucoma	Under glaucomatous stress conditions, Cav-1is involved in the negative regulation of BDNF/TrkB survival signaling	Negative association of Shp2 on BDNF/TrkB signaling is effectively dependent on the Cav-1 protein and provides an in vivo protective effect under experimental glaucoma conditions.	[125]
Cav-1-/- mice	Normal condition	Loss of Cav-1 may influence vascular dysfunction and reduced RGC signaling in the absence of structural loss.	The absence of Cav-1 leads to defective neurovascular coupling at the optic nerve head, ocular hypertension, and increased vessel density.	[30]

POAG: primary open-angle glaucoma, AOH: acute ocular hypertension

### 5.5. Role of Caveolin in mediating Shp2 interactions

**Figure 3.**
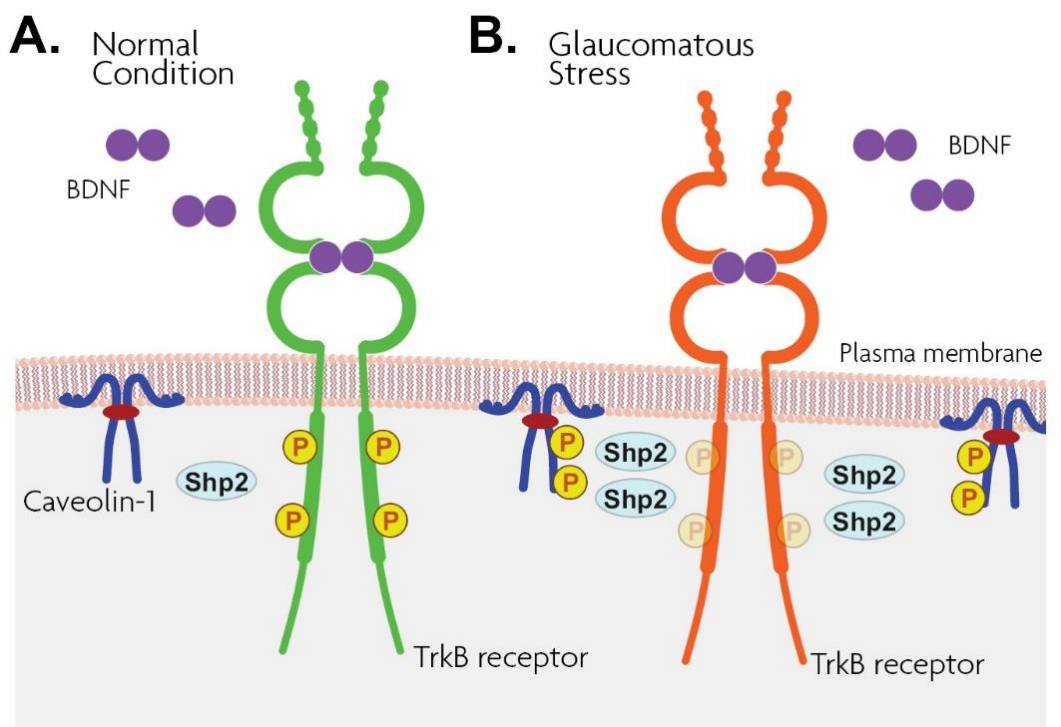
Schematic illustrations of BDNF and its high-affinity receptor TrkB in the RGC membrane. Cav-1, has functional domains which act as a signaling platform for SH2 domain-containing tyrosine phosphatase-2, Shp2. Shp2 phosphatase increasingly dephosphorylates and deactivates TrkB receptor under stress conditions. (A) under normal conditions BDNF stimulates TrkB phosphorylation and the subsequent downstream signaling, while (B) under glaucomatous stress conditions Shp2 dephosphorylate TrkB and this process is mediated by Cav-1 adaptor protein which helps in translocating Shp2 in the vicinity of its target TrkB receptor.

Transmembrane Cav-1 is an adapter protein that interacts and compartmentalizes a variety of proteins to modulate cellular pathways [111]. To perform this role, caveolins are believed to provide a platform and recruit the cellular intermediates in the caveolae, and mediate the downstream signaling [119, 152]. Under oxidative and hyperosmotic stress conditions, caveolin is shown to become hyperphosphorylated [153, 154]. The Tyr14 phosphorylation status of caveolin is precisely regulated in normal cells and may be used as a docking site for SH2 domain signaling proteins [111, 155]. Cav-1 hyperphosphorylation, however, has also been previously demonstrated in RGCs [84], Astrocytes [156], and neurons [157] under stress conditions while in cultured cell lines derived from the trabecular meshwork of glaucoma patients, treatment with dexamethasone, the phosphorylation level of this protein was reduced [158], suggesting that Cav-1 phosphorylation under different circumstances is mediated in a cell-specific manner and that in glaucomatous conditions it is possibly restricted to RGCs. According to our study, in an induced stress condition (optic nerve axotomy), Cav-1 showed several folds upregulated binding to non-transmembrane SH2 domain-containing phosphatase, Shp2, resulting in activation of the phosphatase which in turn negatively dephosphorylated TrkB receptor (Fig. 3). The prolonged dephosphorylation of TrkB results in loss of axonal integrity and reduced cellular survival through the BDNF-mediated MAPK/Erk signaling cascade [84]. It was proposed that upon the optic nerve injury, transmembrane Cav-1 undergoes significant hyperphosphorylation that is associated with increased activity and binding to Shp2. Caveolin was suggested to mediate the phosphatase action by binding to the Shp2 SH2 domain to relieve the allosteric constraints that sustained the Shp2 auto-inhibited condition [159] and facilitate its activation. Alternatively, it is likely that caveolin may recruit Shp2 in raft microdomains as a platform through which it translocates the Shp2 in the vicinity of its target TrkB receptor for the interaction [160, 161]. Interestingly, the Shp2/TrkB crosstalk was remarkably interrupted when Cav-1 expression was reduced using antisense oligonucleotides (AONs) in RGCs [84, 156]. Dephosphorylation of TrkB through Caveolin-associated effects in RGCs results in prolonged TrkB receptor deactivation. Persistent deactivation of TrkB receptor under stress conditions not only hampers the axonal regeneration and other neuroprotective effects of BDNF and NT4 [161] but also at least in part explains why the protective effects of BDNF therapy on RGC survival following experimental glaucoma conditions have only transient outcomes.

The interaction between Cav-1 and Shp2 has also been investigated in other cells. In astrocytes, Cav-1 was shown to modulate the H2O2-dependent phosphorylation of Shp2 and the subsequent activation of the ERK cascade. The oxidative stress driven by H2O2 stimulates the activity of Shp2, which dominantly depends on the presence of lipid rafts and Cav-1 in particular.

However, any alterations in the expression pattern of lipid rafts such as using lipid raft disrupting agents (filipin III) could suppress the Cav-1-dependant Shp2 activation and subsequent ERK phosphorylation [156]. Moreover, the oxidative stress primarily induces the phosphorylation of Cav-1 which facilitates the association between the two proteins while importantly a phosphorylation-ablated form of Cav-1 was not able to bind to Shp2 and significantly reduced the extent of Shp2 activity [111]. These results emphasize that caveolin-1 in different cell types and in facing different situations may perform distinct physiological functions.

## 6. Conclusion and future direction

Currently, the only available form of treatment for glaucoma patients is reducing the IOP level either through medications or surgery. Importantly, for some patients, this management may not be adequate, and they continue to present with disease progression leading to the gradual loss of vision. Consequently, unravelling the IOP-independent mechanisms that are relevant to disease pathophysiology may provide new opportunities to assess and treat glaucoma. This requires a comprehensive understanding of the neurodegenerative pathways underlying RGC death. In this review, we discussed various mechanisms of ganglion cell injury in glaucoma among which neurotrophin deprivation plays an important function. Furthermore, genetic defects might also be associated with the pathogenesis of this disease and the discovery of the *CAV-1* gene locus has been shown to confer an increased risk of glaucoma. The role of Cav-1 in the regulation of various cell signaling pathways has previously been well established although its implication in ocular functions relevant to ocular pathophysiology has only recently been appreciated. It has been shown that in the retina and under stress conditions, this gene is involved in the negative regulation of BDNF/TrkB survival signaling as one of the main pathways in maintaining the health of RGCs. BDNF or TrkB receptor gene therapy has been investigated to prevent RGC death as a neuroprotective strategy. However, these could only provide transient protection to RGCs under elevated IOP conditions and eventually, the ganglion cells succumb to the degenerative cascade of pathological processes. Recent data indicated that this might be at least in part due to *in vivo* interactions of Cav-1 with Shp2 phosphatase in the inner retina and their role in regulating TrkB signaling and mediating the integrity of inner retinal function in glaucoma conditions. Based on these studies, Shp2 negatively impacts the regulation of this pathway which is effectively dependent on the Cav-1 protein, although the precise mechanism of the *in vivo* interactions and their function remained unknown. Therefore, understanding the intricate interactions between BDNF, TrkB, Shp2, and caveolin proteins holds significant promise for the development of novel therapeutic strategies to halt or delay disease progression and ultimately improve the clinical management of glaucoma patients. Targeting this pathway could potentially enhance neuronal survival, protect against optic nerve damage, and mitigate visual impairment. The review summarises these two molecules (Cav-1 and Shp2) as potential targets for neuroprotective therapy in glaucoma and potentially other neurodegenerative disorders. Indeed, further detailed studies will be needed to gain a complete understanding of the mechanisms involved in glaucoma pathogenesis.
